# Physiologically informed organismal climatologies reveal unexpected spatiotemporal trends in temperature

**DOI:** 10.1093/conphys/coae025

**Published:** 2024-05-16

**Authors:** Aubrey Foulk, Tarik Gouhier, Francis Choi, Jessica L Torossian, Allison Matzelle, David Sittenfeld, Brian Helmuth

**Affiliations:** Department of Marine and Environmental Sciences, Northeastern University Marine Science Center, Nahant, MA 01908, USA; Department of Marine and Environmental Sciences, Northeastern University Marine Science Center, Nahant, MA 01908, USA; Department of Marine and Environmental Sciences, Northeastern University Marine Science Center, Nahant, MA 01908, USA; Department of Marine and Environmental Sciences, Northeastern University Marine Science Center, Nahant, MA 01908, USA; Volpe Center, U.S. Department of Transportation, Cambridge, MA 02142, USA; Department of Marine and Environmental Sciences, Northeastern University Marine Science Center, Nahant, MA 01908, USA; Center for the Environment, Museum of Science, Boston, MA 02114, USA; School of Public Policy and Urban Affairs, Northeastern University, Boston, MA 02115, USA; Department of Marine and Environmental Sciences, Northeastern University Marine Science Center, Nahant, MA 01908, USA; School of Public Policy and Urban Affairs, Northeastern University, Boston, MA 02115, USA

**Keywords:** Body temperature, forecasting, heterogeneity, intertidal, quantile regression, thermal stress**Abbreviations:** RBM, robomussel; WS, weather station; CFSR, Climate Forecast System Reanalysis; OLS, ordinary least squares

## Abstract

Body temperature is universally recognized as a dominant driver of biological performance. Although the critical distinction between the temperature of an organism and its surrounding habitat has long been recognized, it remains common practice to assume that trends in air temperature—collected via remote sensing or weather stations—are diagnostic of trends in animal temperature and thus of spatiotemporal patterns of physiological stress and mortality risk. Here, by analysing long-term trends recorded by biomimetic temperature sensors designed to emulate intertidal mussel temperature across the US Pacific Coast, we show that trends in maximal organismal temperature (‘organismal climatologies’) during aerial exposure can differ substantially from those exhibited by co-located environmental data products. Specifically, using linear regression to compare maximal organismal and environmental (air temperature) climatologies, we show that not only are the magnitudes of body and air temperature markedly different, as expected, but so are their temporal trends at both local and biogeographic scales, with some sites showing significant decadal-scale increases in organismal temperature despite reductions in air temperature, or vice versa. The idiosyncratic relationship between the spatiotemporal patterns of organismal and air temperatures suggests that environmental climatology cannot be statistically corrected to serve as an accurate proxy for organismal climatology. Finally, using quantile regression, we show that spatiotemporal trends vary across the distribution of organismal temperature, with extremes shifting in different directions and at different rates than average metrics. Overall, our results highlight the importance of quantifying changes in the entire distribution of temperature to better predict biological performance and dispel the notion that raw or ‘corrected’ environmental (and specially air temperature) climatologies can be used to predict organismal temperature trends. Hence, despite their widespread coverage and availability, the severe limitations of environmental climatologies suggest that their role in conservation and management policy should be carefully considered.

## Teaser

Trends (climatologies) of environmental conditions often focus on changes in averages. Using records of animal body temperature, we show hidden trends in how extreme conditions are not always reflected in trends in average temperatures, and animal temperatures do not closely track changes in air temperatures.

## Introduction

A large body of literature has documented how global climate change acts at regional and local scales to drive ecological patterns that are inherently heterogeneous in both space and time. The complex nature of these interactions between global climate-driven stressors and smaller-scale modifiers is well documented in terrestrial (Schulze, 2000; Ashcroft *et al.,* 2009; Carroll *et al.,* 2016), freshwater (Palmer *et al.,* 2010; Tonolla *et al.,* 2012) and marine (Lima and Wethey, 2012; Dias *et al.,* 2018; Nolan *et al.,* 2021; Starko *et al.,* 2022) habitats and has critical implications for how we manage and protect ecosystems (Hall *et al.,* 2016). While these heterogeneities manifest in nature across numerous environmental drivers, one of the most obvious—and the focus of this study—is temperature, which is universally recognized as one of the most important components of global climate change because of its biological effects (Sears *et al.,* 2016). Early studies that focused on documenting broad spatiotemporal trends in climate impacts often used coarse-grained (e.g. latitudinal, annual) information in both drivers and biological responses (Parmesan, 1996) as proxies for ecological processes across multiple scales. Unfortunately, these trends based on simplifying assumptions often masked meaningful variability at biologically relevant scales and thus yielded spurious altitudinal or latitudinal ‘gradients’ in temperature that later studies revealed to instead be cryptic geographic mosaics (Helmuth *et al.,* 2006a; Kroeker *et al.,* 2016; Adams *et al.,* 2020; Pinsky *et al.,* 2020; Vye *et al.,* 2020; Wang *et al.,* 2020b; Beaty *et al.,* 2023).

To avoid the emergence of such spurious patterns, more recent work has emphasized the need to better match the spatial and temporal scales of environmental drivers to those of biological processes (Stenseth *et al.,* 2002; Gunderson *et al.,* 2016; Pincebourde *et al.,* 2016; Miller and Dowd, 2019). Such an approach can promote our ability to predict organismal performance (Pincebourde and Woods, 2020) by capturing short-term and geographically localized extreme conditions associated with events such as heat waves (Maxwell *et al.,* 2019; Amaya *et al.,* 2023) or cold snaps that would be smoothed or averaged-out out by traditional coarse-scaled products. The nascent but rapidly expanding field of Conservation Physiology has played a key role in this endeavour by documenting the variability of both environmental drivers at fine scales and the sensitivity of organisms to those conditions (Cooke and O’Connor, 2010; Tomlinson *et al.,* 2018; Cooke *et al.,* 2021; Seebacher *et al.,* 2023).

A critical consideration that is often overlooked in these discussions, especially with studies exploring biogeographic responses to climate change, is the extent to which trends in environmental conditions such as air temperature are truly diagnostic, in even relative terms, of biological response. While it has long been recognized by thermal biologists that organismal (body) temperature drives physiological responses such as performance or survival, these body temperatures (‘operative temperatures’) cannot be assumed to be equivalent to environmental (air or water) temperature (Porter and Gates, 1969; Bakken, 1992; Kearney, 2006; Pincebourde *et al.,* 2016). For instance, in terrestrial (and, during low tide, coastal intertidal) environments, solar radiation serves as the primary heat source for both habitat and organisms (Porter *et al.,* 1973). This heat is then mixed within the overlying boundary layer so that air temperatures measured even a few meters above the substrate can be radically different from what organisms are experiencing. To add further complexity, organisms also influence heat exchanges based on their size, colour and material properties, so that two organisms living side by side can experience radically different body temperatures even when exposed to the same microclimatic conditions (Porter and Gates, 1969). During aerial exposure at low tide, for example, intertidal mussels can achieve body temperatures that are upwards of 15°C hotter than the surrounding air (Denny *et al.,* 2011), while an adjacent seastar may have a body temperature cooler than air temperature because of loss of heat through evaporation (Broitman *et al.,* 2009).

Acknowledging and quantifying the physical mechanisms that ultimately drive body temperatures of an organism thus creates a considerable conundrum for how we use commonly collected data, such as air temperatures from weather stations (WS) or buoys, to make predictions of biological processes (Kearney, 2006; Seebacher *et al.,* 2023). These complexities all suggest that the source of the data can significantly influence predictions of spatial and temporal patterns in the vulnerability of organisms in nature (Bütikofer *et al.,* 2020). For instance, Mislan and Wethey (2011) showed that biophysical models driven by a series of gridded weather data differed not only from the baseline WS and from each other, but the ‘best’ models for a given outcome were also not consistent between geographic sites. These complexities are particularly pertinent when attempting to forecast the likely impacts of global climate change over large biogeographic and/or broad temporal scales. Specifically, while the most common indicators of global climate change are trends in air, land surface, or sea surface temperatures, to what extent are these trends (i.e. climatologies, sensu NOAA National Centers for Environmental Information) in environmental drivers truly diagnostic of changes in organismal physiological performance and mortality risk, when we know that air temperatures are not equivalent to body temperature?

Multiple authors have used heat budget models that predict the body temperatures of organisms using environmental data (Porter and Gates, 1969; Mislan *et al.,* 2014; Maclean *et al.,* 2019; Kearney and Porter, 2020; Wethey and Woodin, 2022; Buckley *et al.,* 2023) and have used these approaches to infer trends in physiological response in space and time (e.g. Kearney *et al.,* 2008). Fewer studies have deployed biomimetic sensors (Judge *et al.,* 2018)—self-contained data loggers which match thermal characteristics of an organism in question and thus record temperatures that are close approximations of body temperature (Lima and Wethey, 2009; Seabra *et al.,* 2011; [Bibr ref41][Bibr ref41], 2016). Here, we leverage one of these datasets—biomimetic sensors of intertidal mussels spanning the US western coastline (Helmuth *et al.,* 2017)—to explicitly test the hypothesis that temporal trends in environmental (air) temperature are effective as a relative indicator of trends in animal temperature. The intertidal zone serves as an excellent model system to study global change effects as resident organisms are repeatedly exposed to dynamic variation in both terrestrial and marine environmental conditions, making them particularly sensitive to thermal stress (Connell, 1972; Wethey, 1986).

We evaluate local and biogeographic long-term trends in biomimetic temperatures, henceforth referred to as ‘organismal climatologies’ (Helmuth *et al.,* 2010), using analysis of simple trends and compare these to patterns revealed using air temperature. Notably, we use the term climatology to refer to temporal trends in a specific parameter and argue that just as there are climatologies for environmental parameters such as air temperature (Wang *et al.,* 2020a), surface winds (Laurila *et al.,* 2021), or sea surface temperature (Hu *et al.,* 2021), the exploration of climatologies of a species’ body temperature can lend insights into how climate change affects organism physiology.

A further consideration is how large data sets are analysed and specifically if and when temporal and spatial averages are indicative of the incidence of shorter-term events. Solely focusing on changes in mean conditions can overlook important signals occurring in other parts of the statistical distribution. For example, temporal means may hide the occurrence of ecologically significant extreme events like heat waves (Sunday *et al.,* 2019) or, in space, the presence of microrefugia (Benedetti-Cecchi *et al.,* 2003; Salois *et al.,* 2022). Various statistical approaches have improved our ability to evaluate temporal changes in the entire distribution of ecological and environmental variables more thoroughly, oftentimes revealing trends that differ in magnitude and direction from those observed for the mean. For instance, temporal trends in upwelling event duration along the US west coast exhibited more moderate increases for extreme than intermediate quantiles at some latitudes, while at others, the relatively low quantiles showed comparatively stronger increases (Iles *et al.,* 2012). Similarly, trends in the extreme high and low quantiles differed from each other, and from the mean, in European (Matiu *et al.,* 2016) and Australian (Duan *et al.,* 2022) temperature records, illustrating how mean temperatures potentially hide relevant thermal variation that may be responsible for ecologically significant processes (Camacho *et al.,* 2015).

Therefore, we furthermore compare trends of body (biomimetic) temperature across the entire statistical distribution, elucidating patterns in extremes potentially hidden by coarse-scale averaging. Overall, our analyses reveal that (i) spatiotemporal trends vary idiosyncratically across the entire temperature distribution and (ii) environmental climatologies cannot be used as accurate proxies for organismal climatologies at any spatial scale. Taken together, these results suggest that using environmental proxies to predict biological performance may yield substantial mistakes.

## Materials and Methods

### Biomimetic temperature datasets

Organismal body temperature records were mined from an existing biomimetic sensor database (Helmuth *et al.,* 2017). Hereafter referred to as ‘robomussels (RBMs)’, these biomimetic sensors are capable of mimicking (±1.5–2°C of living animals; Fitzhenry *et al.,* 2004) the thermal properties—including colour, shape, mass and material—of the ecologically important species *Mytilus californianus*, the California mussel. By logging temperatures at scales relevant to organisms—driven by a combination of local weather interacting with microhabitat and organism morphology—RBMs enable a direct link between field observations of exposure to physiological sensitivity.

**Figure 1:**
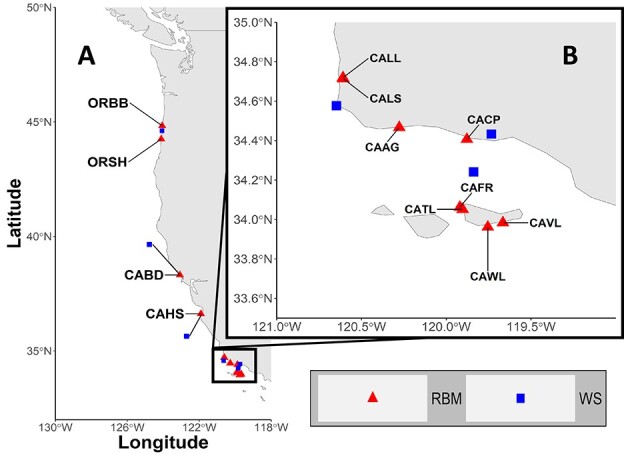
Map of the (**A**) full study region and (**B**) inset of the southern portion of the study region showing RBM (red triangles) and WS (blue squares) sites.

RBMs were deployed at 12 sites along the US Pacific Coast (Fig. 1; red triangles): 2 sites in Oregon [Boiler Bay (ORBB) and Strawberry Hill (ORSH)] and 10 sites in California [Bodega (CABD), Hopkins (CAHS), Lompoc Landing (CALL), Lompoc South (CALS), Alegrıa (CAAG), Coal Oil Point (CACP), Fraser (CAFR), Trailer (CATL), Valley (CAVL) and Willows (CAWL)]. Each of the 12 field sites were characterized by numerous microsites (i.e. individual loggers; *n* = 3–11 with one to six active loggers at any given time step) located on moderately wave-exposed shorelines. RBM loggers were deployed in growth position (posterior upward) on hard rock substrate within intact mussel beds at ‘mid-tidal’ level [i.e. mean lower low water (MLLW) +1.5–1.7 m]. Sites on horizontal (upwards facing) substrata with unimpeded skyview factor were selected in order to standardize across sites (see more details in Helmuth *et al.,* 2006a). Analyses from previous studies on RBMs from this geographic region indicated that variability between microsites at any given site was minimal when instruments were deployed in regions of similar wave exposure and tidal elevation and attached to substrata with uniform slope and aspect (Gilman *et al.,* 2006a). Therefore, microsites within a site were treated as replicates and averaged at each time step to ultimately generate one time series per site. Deployments were active from 2000 to 2015 with temperature measurements recorded at 10-minute intervals. Deployment durations for individual microsites varied from site to site, with interruptions due to instrument loss or damage; however, combined records for each site remained relatively continuous throughout the entire study period.

### Environmental datasets

Two different air temperature datasets were curated and matched to each of the 12 RBM sites. The first set was gathered from local WSs (*n* = 6), all of which met the National Weather Service (NWS) site and exposure standards and were located less than 40 km from a paired site (Fig. 1; blue squares). Stations were terrestrially based, with the exception of one marine buoy, which was exclusively paired to the sites located on the Channel Islands. Sampling intervals ranged from hourly to daily time steps. The second set was sourced from the National Center for Environmental Prediction (NCEP) Climate Forecast System Reanalysis (CFSR). This coupled atmosphere–ocean–land surface–sea ice system generates datasets at high resolution with global coverage (Saha *et al.,* 2010). Modelled data were available at approximately 38-km grid resolutions and were downloaded as maximum temperatures at daily time steps.

### Time series preparation

For each of the three datasets at each site, temperature time series were sourced at, or upscaled to, daily maximum temperatures. Time series were then temporally limited to the months of April through September (summer). These constraints isolated a physiologically relevant metric of high temperature while simultaneously removing the complication of separating water (high tide) and aerial (low tide) temperatures recorded by the RBMs, as summertime daily aerial exposures are when *M. californianus* experience the highest body temperatures (Helmuth *et al.,* 2002). Seasonal temporal limitations were also cross-checked with previous analyses to ensure that the months historically recording the highest body temperatures throughout the entire geographic extent of sites were included in the analyses (Helmuth *et al.,* 2006a). For comparative purposes, time series were also truncated to days in which values were present for all three data sources. While sampling frequency can certainly influence the time series, because of our interest in trends rather than discrete events, these bias effects should be largely nullified.

### Statistical analyses

To determine average trends in temperature change over time, ordinary least squares (OLS) regressions were performed on organismal and environmental time series at each of the geographic sites. Focusing on summary statistics, here the mean trend in daily maximum temperature, can often conceal more complex patterns associated with extremes. Hence, to complement our OLS analysis focusing on mean trends, we also conducted quantile regression (Koenker and Bassett, 1978; Cade and Noon, 2003) to provide a more complete understanding of the temporal shifts in the entire distribution of temperature at each site. Specifically, we used quantile regression to estimate temporal trends across 100 evenly spaced temperature quantiles ranging from τ = 0.01 to τ = 0.99 for organismal datasets at each of the 12 sites. Regressions were ultimately limited to the 5th–95th percentiles (i.e. τ = 0.05 to τ = 0.95) to avoid unreliable estimates associated with high uncertainty due to low sample sizes and outliers. All analyses were performed in the R version 4.2.2 programming environment, using the package quantreg version 5.94 (Koenker, 2005) for quantile regression analyses.

## Results

### Incongruent organismal and environmental local temperature trends

**Figure 2:**
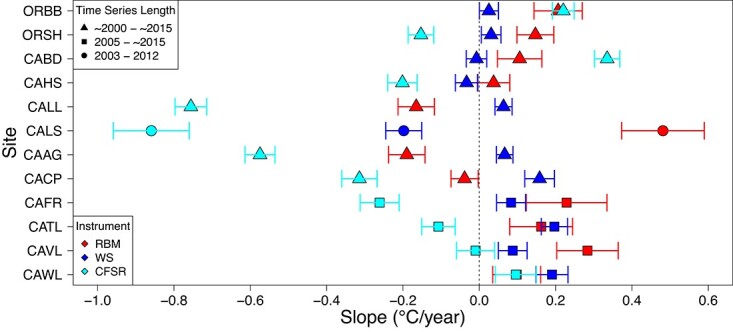
The rate of change (slope; °C/year) and 95% confidence intervals generated by OLS regressions run on the RBM (red), WS (dark blue), and CFSR (light blue) temperature time series from 2000–2015. Divergent time series lengths are denoted by shape (triangles = ~2000–~2015, squares = 2005–~2015, and circles = 2003–2012). A dashed vertical line is placed at zero as a visual cue of positive (>0) and negative (<0) trends. Sites are ordered latitudinally from the northernmost site (i.e. Oregon; top) to the southernmost site (i.e. Southern California; bottom).

Daily maximum temperatures exhibited high within-site variation across data sources, as expected, with extremes for RBMs (i.e. organismal body temperatures) generally greatly exceeding the magnitude and the range of local WS or model-derived (CFSR) air temperatures (Supplementary Fig. S1.1–S1.4). These differences were most prominent at high- (WS & CFSR) and low- (WS) latitude sites (Supplementary Fig. S1.1). OLS analysis indicated that rates of temperature change over time (°C/year) produced from the three data sources over the period 2000–15 also often differed in magnitude and/or direction (Fig. 2; Supplementary Table S1). For example, at site ORSH (Strawberry Hill, OR), the RBM and WS datasets showed positive trends in daily maximum temperature change over time, while the CFSR data showed a negative trend. Directional differences in trends are potentially most troubling when environmentally and organismally derived trends are contradictory (e.g. Fig. 2—CATL, CAAG, CALS). Even when the sign of the temporal trends in temperature was congruent across the environmental and organismal datasets (e.g. at ORBB or CAWL), the magnitude of the change, and ultimately the corresponding change in risk of physiological stress incurred, could still vary greatly. Overall, the incongruent local (within-site) temporal trends observed across the environmental and organismal temperature products suggests that predicting trends in physiological performance or survival from even highly ‘downscaled’ environmental data (air temperature) is likely to yield major mistakes.

### Incongruent organismal and environmental biogeographical temperature trends

OLS analysis of RBM data indicated that rates of temperature change over time (°C/year) were variable along the coastline of the western US (Fig. 2; Supplementary Table S1) and did not conform to any latitudinal gradient. Higher- and lower-latitude sites showed patterns of increasing daily maximum RBM temperatures over the time periods examined (2000–15), while mid-latitude sites showed predominantly decreasing trends over time, all with varying rates of change. A lack of a strong unidirectional latitudinal trend in rates of temperature change was also apparent in the WS and CFSR air temperatures across the same period. WS datasets produced the mildest temperature trends over time (closest to a zero slope), while CFSR datasets generated primarily negative trends over time with steeper slopes. Most importantly, the relationship between the trends produced by different data sources did not hold across sites (Fig. 2; Supplementary Table S1). This dynamic response could be particularly problematic for ecological forecasting, as both correlative and biophysical approaches assume that the relationship between organismal and environmental temperature is stationary or consistent in time. While quantitative differences between the data products within local sites were anticipated, the incongruent qualitative patterns observed at biogeographical scales highlight the dangers of using environmental proxies instead of organismal temperature at any scale.

### Inconsistent changes in the distribution of organismal temperature

**Figure 3:**
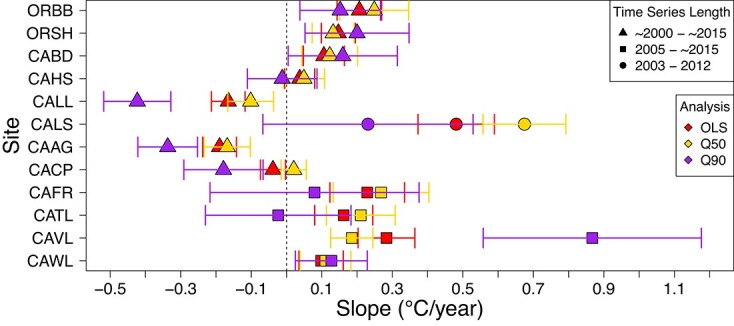
The rate of change (slope; °C/year) and 95% confidence intervals generated by OLS (red) and quantile regressions (Q50: 50th percentile, τ = 0.50 in gold and Q90: 90th percentile, τ = 0.90 in purple) run on RBM temperature time series from 2000 to 2015. Divergent time series lengths are denoted by shape (triangles = ~2000–~2015, squares = 2005–~2015, and circles = 2003–2012). A dashed vertical line is placed at zero as a visual cue of positive (>0) and negative (<0) trends. Sites are ordered latitudinally from the northernmost site (i.e. Oregon; top) to the southernmost site (i.e. Southern California; bottom).

**Figure 4:**
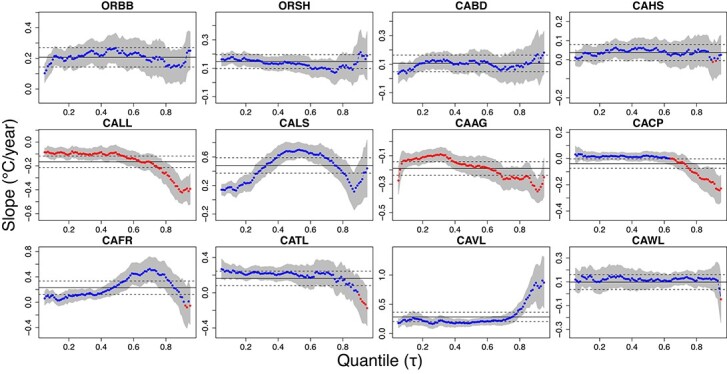
Quantile regression analysis run on the RBM time series from 2000 to 2015 at each of the 12 sites. Blue (red) data points represent positive (negative) rates of change (i.e. slope; °C/year) from the 5th to the 95th percentile. Grey-shaded areas represent the 95% confidence intervals of the quantile regression. Black solid and dashed lines represent the slopes and associated 95% confidence intervals, respectively, as determined by corresponding OLS analyses. Sites are ordered latitudinally across rows from north (top left) to south (bottom right). Note the differences in axes scale so that intra-site, rather than inter-site, comparisons are more distinctly visualized.

Results from the OLS analyses identified that trends in temperatures of the surrounding environment (WS and CFSR) were inherently different from those simulating organismal body temperatures (i.e. RBMs). While we acknowledge that the differences in RBM and ‘environmental’ data source trends are also conflated with spatial position (i.e. the paired WS and RBM datasets were not obtained at exactly the same geographical location), the marked distinction is worth noting as the datasets explored at each site have traditionally been used interchangeably. To further investigate trends most relevant to the organism, the following extended analyses were limited to the RBM datasets. Specifically, quantile regression analyses were performed on RBM temperature time series from each of the 12 sites (Figs 3 and 4). It should be noted that rates of change derived from the OLS and the τ = 0.5 quantile regressions (i.e. the median or 50th percentile) are slightly different because although they both represent central tendencies, they are calculated with respect to the mean and median, respectively. As with the OLS results described above, trends obtained from distinct quantiles often differed in terms of magnitude or sign, and these discrepancies varied geographically across sites (Supplementary Table S2, Figs 3 and 4). Conflicting trends in the 50th and 90th percentiles are of particular ecological significance, as they suggest that the central tendencies and extremes of temperature experienced by organisms are changing at different rates (Fig. 3). Inter-site comparisons showed some compelling differences, with mid-latitude sites such as CACP, CAAG and CALL showing negative trends in the high quantiles, whereas other mid-latitude sites showed positive trends across all quantiles (especially the high ones; see CAVL). Altogether, this extensive spatiotemporal heterogeneity in temperature trends highlights the importance of understanding whether organismal performance and survival, and subsequently range shifts, are primarily driven by mean or extreme temperatures.

## Discussion

With the availability of remote sensing products and widespread deployments of stationary weather instruments, it has become commonplace to use environmental datasets to infer organismal physiological responses or at least to predict trends in space and time. However, our results clearly show that we cannot rely on these traditional methodologies to predict spatiotemporal patterns of climate change induced stress. Although previous studies have shown that quantitative differences exist between organismal and environmental data sources, we found that even qualitative trends in temperature over a 15-year time period dissociate at both local and biogeographical scales and that the correlative relationships between instruments are inconsistent across sites. Specifically, in many cases, while air temperatures suggested little or no climatic trends at local levels, measurements relevant to organismal biology showed dramatic warming. At other sites, the reverse was true. Hence, monitoring and predicting the impacts of climate change on ecosystems will require shifting away from environmental proxies and toward obtaining a more complete, nuanced understanding of how individual organisms experience, and physiologically respond to, environmental variation, i.e. organismal climatologies. Furthermore, even looking only at organism temperatures, different portions of the temperature distribution shift in distinct ways over time at both local and regional scales. Thus, trends in mean conditions are not necessarily diagnostic of trends in extremes.

### Why commonly used temperature proxies fail to predict physiological performance

Our results indicated great variation in temperature trends that were highly dependent on their data source. This has important implications for management and conservation as trends based on instruments most relevant to organismal physiology differ from those obtained from more traditional and widespread data sources. Why climatologies based on RBMs differ from those based on air or land surface temperatures remains an open question. Previous modelling studies have suggested that extremes in air temperature are needed for, and therefore may be diagnostic of, extremes in body temperature (Gilman *et al.,* 2006b; Mislan *et al.,* 2014), although the ability of air temperature to serve as an effective proxy for body temperature has also been shown to sharply decrease once temperatures exceed the ‘optimal’ temperature for an organism (Denny *et al.,* 2011; Kish *et al.,* 2016). However, these models also assumed cloud-free, clear sky conditions were a prerequisite of extreme temperatures, which may not always be the case. Any decadal-scale trends in cloud cover or coastal fog could potentially explain why trends in mussel temperature were not matched by air temperature (Johnstone and Dawson, 2010; Williams *et al.,* 2018). Similarly, any changes in wave splash (Harley and Helmuth, 2003) or wind speed (Helmuth *et al.,* 2011) would also result in changes in body temperature not reflective of changes in air temperature. Finally, the timing of mid-day low tide has been shown as a strong determinant of latitudinal patterns in mussel temperature (Helmuth *et al.,* 2002) and these are known to change over decadal scales (Denny and Paine, 1998). Likely, it is some combination of the above factors that contributes to the patterns observed, but the causal drivers remain to be determined. This argues strongly against the use of air temperature alone as even a relative proxy for animal temperature, especially given that climate change is driving shifts in multiple drivers such as wind, cloud cover and (in coastal systems) wave heights (Young *et al.,* 2011; Qu *et al.,* 2014; Sydeman *et al.,* 2014).

### Importance of scale

The scales at which we sample and assess the environment can significantly affect our perceptions of climatic, and ultimately ecological, patterns and trends (Peterson *et al.,* 1998; Schoch *et al.,* 2006; Montalto *et al.,* 2014; Brito-Morales *et al.,* 2018; Salois *et al.,* 2022). Microclimates and other measures of small-scale environmental heterogeneity are increasingly being recognized as important drivers of ecosystem dynamics (Potter *et al.,* 2013; Choi *et al.,* 2019). For example, Kearney (2006) delineates the ‘niche’ temperature as ‘a subset of those environmental conditions which affect a particular organism’, from aspects of the ‘environment’ such as local air temperature. However, the consequences of fine-scale heterogeneity are often ignored when exploring trends over large geographic scales. For instance, a common assumption in climate change literature is that extinction risk due to high temperature exposure should be highest towards a species’ equatorial range edge that then decreases along a ‘gradient’ moving poleward. As global conditions continue to warm, extinctions at the equatorial range edge are expected to cause a poleward range shift. However, the results of our study show contradicting patterns with several southern (northern) sites characterized by negative (positive) trends [i.e. a downshift (upshift) in temperature distribution]. In this scenario, disregarding the underlying spatial variability in temperature trends may result in mismanagement through misidentification of climate-related extinction risk. This instead argues for a place-based management strategy that moves beyond overly simplistic assumptions of latitudinal gradients.

The role of spatial heterogeneity could also explain why the abundant distribution hypothesis, which states that the density and demographic performance of species should be highest in the center of their geographical range (Brown, 1984), has come under increased scrutiny (Beaty *et al.,* 2023). Recent literature reviews exploiting large databases across numerous species groups (Santini *et al.,* 2019) have contested the idea, primarily based on a consistent lack of empirical support across distinct sampling protocols. Instead, they conclude that the concept is an oversimplification of complex, multiscale biogeographical forces generating irregular spatial abundance patterns. Our results support this conclusion and suggest that a narrow focus on range shifts only at geographic distribution margins may overlook ecologically important population fluctuations, or even local extinctions, at sites well within a species range (Beaty *et al.,* 2023). This has important implications for management efforts, as decision-makers responsible for communities on landscape scales may need to account for varying pockets of thriving or vulnerable populations. Specifically, if we assume organisms are assembled in predictable patterns over large-scale spatial gradients, we may fail to account for areas of spatial refugia (Pulliam, 1988) that may serve as source populations for surrounding ecosystems or fail to recognize that a focal system or location is in fact a sink relying on neighbouring areas to remain populated. Therefore, understanding the underlying mosaic patterns across scales is key for identifying sources and sinks, which are key components to many conservation and management efforts (Gouhier *et al.,* 2010; Menge *et al.,* 2011; Hannah *et al.,* 2014).

Temporal scales can also impact our perception of climate change. A rapidly expanding literature is exploring extreme climate events (Van De Pol *et al.,* 2017; IPCC, 2023) such as heatwaves. In both terrestrial and aquatic environments, heatwave frequency, duration, spatial extent and magnitude have increased globally in the last century (Oliver *et al.,* 2018; Smale *et al.,* 2019), a trend that is expected to continue under current warming (Oliver *et al.,* 2019). When defining a heatwave, spatiotemporal scale is of the utmost importance (Hobday *et al.,* 2016; Amaya *et al.,* 2023), especially when attempting to identify how changes in putative indicators relate to organismal fitness and survival (i.e. its body temperature; Bertolini and Pastres, 2021). The coral bleaching literature has wrestled with this dilemma by defining heat waves based on local climatic norms (Hobday *et al.,* 2016), but still issues arise given the large variation in vulnerability among coral species within a location (Humanes *et al.,* 2022) and the often hard-to-predict variation in responses over depths (Giraldo-Ospina *et al.,* 2020; Wyatt *et al.,* 2023). Similarly, our results suggest that in intertidal systems, heat waves based solely on air temperature may miss extreme events that ‘go under the radar’ because they are not reflected by air temperature. They also strongly suggest that a focus on trends in temporal means may not accurately capture trends in extreme events.

### Shifting away from changes in mean conditions

Large-scale climatic trends often focus on changes in mean conditions, but the temporal and spatial dynamics hidden by changes in the mean (Di Cecco and Gouhier, 2018) are critical to the fitness of organisms. The results of this study suggest that understanding spatial or temporal patterns in the average temperature (or direct metrics of physiological state) of organisms may be far less meaningful than characterizing the full temperature (or performance) distribution and especially that trends in averages are not predictive of trends in extremes. The disparity in these trends is especially large when organismal temperatures are non-linearly translated into estimates of physiological performance. This has been discussed, for example, by studies exploring the consequences of Jensen’s inequality for translating patterns of temperature into patterns of performance (Denny, 2017; Mitz *et al.,* 2017). Changes in temperature quantiles that correspond to the steepest regions of a species’ thermal performance curve would subsequently be expected to lead to the fastest rates of change in performance and, at the highest quantiles, in mortality.

Our results suggested a large amount of spatiotemporal heterogeneity in the way that body temperature distributions are shifting, with contrasting patterns emerging both amongst quantiles within sites and at similar quantiles across sites. We found that patterns in different quantiles were oftentimes fundamentally different than those derived using averages. This was particularly concerning in instances when the highest quantiles were increasing at faster rates than the average, suggesting that periods of most acute stress may be becoming more intense more quickly than anticipated using conventional methods. Since temperature extremes are often key drivers of long-term population and community dynamics (Wethey *et al.,* 2011), it is important to adopt statistical approaches that track trends beyond the mean to gain a better understanding of organismal performance and the emergence of cryptic refugia.

### Moving forward: insights from the perspective of the organism

Recent calls to action continue to prioritize using physiologically relevant data, as opposed to habitat-level measurements, to assess or predict organism responses to various climatic drivers (Gilman *et al.,* 2006b; Denny *et al.,* 2009; Jones *et al.,* 2009; Buckley *et al.,* 2023). Organisms make decisions in response to cues in their immediate environment, as they may have no insight as to where suitable locations could be found on a larger scale. Therefore, organisms may distribute themselves in ways that may appear counterintuitive relative to large-scale gradients, as they navigate the smaller-scale mosaic layout of environmental conditions. As a result, a shift in species distribution is indirectly influenced by both climate variability and climate change via the fine-scale interactions between organisms and their immediate environments—including mosaics and gradients of environmental variables—mediated by a multitude of other factors including physiology, behaviour, evolution and dispersal (Torossian *et al.,* 2016; Pinsky *et al.,* 2020). In the face of environmental change, our ability to predict the environmental conditions experienced by organisms, as opposed to those of the surrounding habitat, is critical for accurately assessing the functioning of ecosystems, and ultimately ecosystem services relevant to society, at scales relevant to managers and practitioners.

Considerations of fine-scale heterogeneities have begun to trickle through when managing ecosystems, for instance by accounting for spatial variability in three dimensions across estuarine models supporting fisheries stock assessments (Yurek *et al.,* 2023), as well as in conservation, where local environmental conditions change more slowly than surrounding areas and populations of organisms are able to survive despite overall warming trends (Morelli *et al.,* 2020; Salois *et al.,* 2022). Associated fine-scale effects manifest both in space (Sears *et al.,* 2016) and time (Kefford *et al.,* 2022), both of which are critical to biologically relevant interpretations. While we have emphasized the utility of data relevant to the scale of the organism, this study did not consider the known high levels of within-site variation in body temperature (Denny *et al.,* 2011) or the differences observed among different species (Broitman *et al.,* 2009). For example, we already ‘spatially homogenized’ the data to some extent by deliberately restricting data collection to microsites with similar tidal elevation, wave splash and solar exposure (sky view factor). We then further treated microsites (i.e. individual loggers) as replicates of a given site, consequently averaging out inter-individual variability within an area pre-defined as a site. Even though microsites were characterized with similar wave exposures, substratum angles and tidal heights, variation in daily maximum temperatures between microsites on any given day could be quite large.

These differences will be exacerbated when considering microsites that differ in tidal elevation, solar aspect and exposure to wave splash (Choi *et al.,* 2019). Denny *et al.* (2011) found that although the mean range of maximal body temperatures among individual RBMs in a single exposed bed was 3.7°C, inter-individual variation in body temperature could be as large as 11.7°C, presumably due to small-scale differences in solar exposure or wave splash. Decreased model skill (i.e. predictive capacity of one RBM for another) shown at high temperatures further emphasizes how localized heat stress events may be (Kish *et al.,* 2016). When considering the additional complications inherent with living organisms, spatiotemporal variability in interindividual physiology, such as thermal tolerance thresholds (Herrando-Pérez *et al.,* 2023), or behavioral choices must also be considered. Similarly, here we only consider temperature, recognizing that the spatial and temporal patterns of multiple stressors almost certainly play a key role in organism fitness (Kroeker *et al.,* 2016). Finally, our results here apply to the temperatures of one species, which, while ecologically important, likely displays patterns in body temperature that differ from other species at the same locations (Broitman *et al.,* 2009).

## Conclusion

The spatiotemporal complexities that underlie interactions between local and global changes emphasize the need to collect and analyse data at scales relevant to the ecosystems and organisms they intend to represent (Benedetti-Cecchi *et al.,* 2003; Helmuth *et al.,* 2006b; Buckley *et al.,* 2018). Although many studies rely on environmental proxies to predict organismal-level processes, these methods often fail to consider the environment as it is experienced and translated into physiological responses. While it has long been understood that the ‘operative body temperature’ of organisms can be radically different from air temperature (Porter and Gates, 1969), this continues to be ignored, especially in coastal ecosystems and especially in studies seeking to quantify trends in space and time (Torossian *et al.,* 2016). This disconnect frequently leads to disjointed scales of physiological responses and environmental measurements or models (discussed in Sears *et al.,* 2011; Boyd *et al.,* 2016), ultimately resulting in incorrect predictions or ecological forecasts.

The recognition and integration of spatiotemporal environmental variability is not only vital to interpreting past and current climate patterns but should also be high-priority considerations when constructing methodologies to predict the ecological impacts of future climate change (IPCC, 2023). The increasing availability of datasets that are collected across large spatial and temporal scales, but which are designed to explicitly be relevant to organism fitness (Seabra *et al.,* 2011), may reveal emerging hotspots of change or stress, which can provide guidance to conservation and management policy and decision makers for where to focus limited time and resources (Seabra *et al.,* 2015). Understanding the implications of using different biological, geographical and temporal scales (Schoch *et al.,* 2006; Bates *et al.,* 2018), and further integrating the result into existing conservation actions, will be vital to understanding and predicting the ecologic and economic impacts of climate change (Seebacher *et al.,* 2023). Organismal climatologies may serve as novel tools useful for bridging the existing gap between environmental trends and organism responses critical to these efforts.

## Supplementary Material

Web_Material_coae025
