# Supplementary material for: Physiologically informed organismal climatologies reveal unexpected spatiotemporal trends in temperature
Source: Conserv Physiol. 2024 May 16;12(1):coae025. doi: 10.1093/conphys/coae025 (PMC11109819; doi:10.1093/conphys/coae025)
Supplement: Web_Material_coae025 [file web_material_coae025.pdf]

Supplementary Material

Table S1: The rate of change (slope; °C/year) and associated standard errors generated by ordinary least squares (OLS) regressions run on the Robomussel (RBM), Weather Station (WS), and Climate Forecast System Reanalysis (CFSR) temperature time series for all 12 sites (rows) from 2000-2015. Significance ($p < 0.05$) is indicated with an asterisk. Blue (red) cells represent positive (negative) trends over time. Sites are ordered latitudinally from Oregon (i.e., north; top) to Southern California (i.e., south; bottom).

Site	RBM	WS	CFSR
ORBB	0.207* ± 0.032	0.025* ± 0.013	0.220* ± 0.014
ORSH	0.147* ± 0.024	0.031* ± 0.013	-0.153* ± 0.017
CABD	0.106* ± 0.029	-0.007 ± 0.014	0.335* ± 0.017
CAHS	0.038 ± 0.021	-0.033* ± 0.015	-0.201* ± 0.019
CALL	-0.165* ± 0.024	0.064* ± 0.011	-0.756* ± 0.021
CALS	0.481* ± 0.054	-0.198* ± 0.024	-0.859* ± 0.050
CAAG	-0.190* ± 0.024	0.067* ± 0.011	-0.574* ± 0.020
CACP	-0.038* ± 0.018	0.158* ± 0.011	-0.314* ± 0.024
CAFR	0.229* ± 0.053	0.083* ± 0.019	-0.261* ± 0.026
CATL	0.162* ± 0.041	0.197* ± 0.017	-0.107* ± 0.022
CAVL	0.284* ± 0.040	0.088* ± 0.019	-0.010 ± 0.025
CAWL	0.098* ± 0.031	0.191* ± 0.021	0.100* ± 0.027

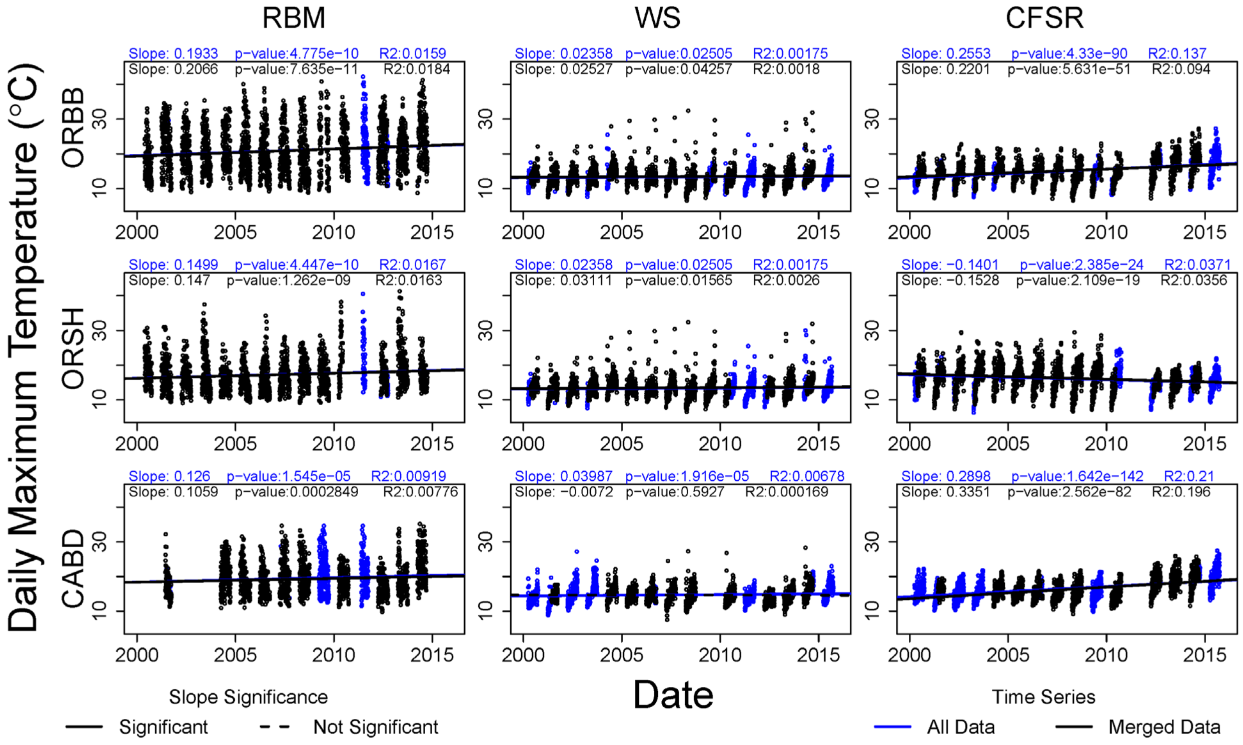

Figure S1.1: Daily maximum temperature time series (black; columns) for Robomussel (RBM; left), Weather Station (WS; center), and modeled (CFSR; right) datasets at three unique sites (rows). Trend lines are generated from simple linear regressions (OLS) run on data simultaneously available at all three data sources (black). For comparative purposes, additional data points and respective trend lines available for each data source (blue) are also shown. Associated R^2 values, p-values, and slopes (°C/year; dashed line if not significant, solid line if significant) are noted at the top of each panel. Sites are ordered latitudinally from north (top) to south (bottom).

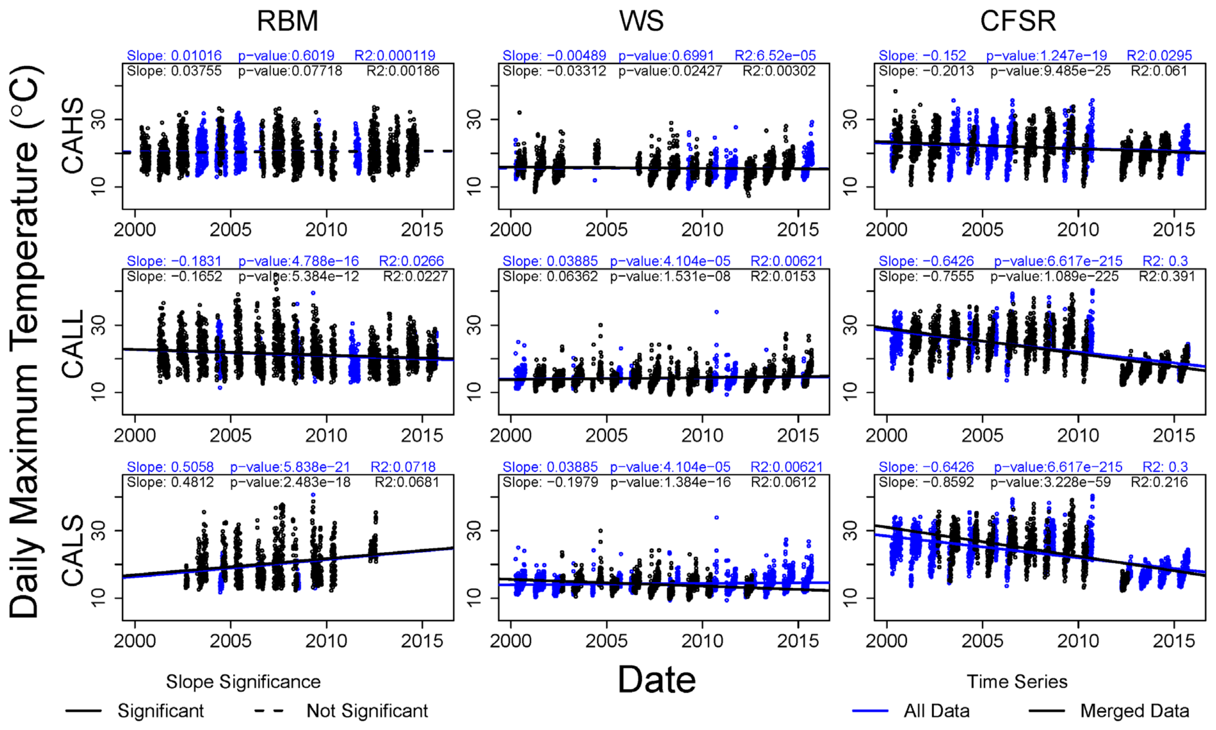

Figure S1.2: Daily maximum temperature time series (black; columns) for Robomussel (RBM; left), Weather Station (WS; center), and modeled (CFSR; right) datasets at three unique sites (rows). Trend lines are generated from simple linear regressions (OLS) run on data simultaneously available at all three data sources (black). For comparative purposes, additional data points and respective trend lines available for each data source (blue) are also shown. Associated R^2 values, p-values, and slopes (°C/year; dashed line if not significant, solid line if significant) are noted at the top of each panel. Sites are ordered latitudinally from north (top) to south (bottom).

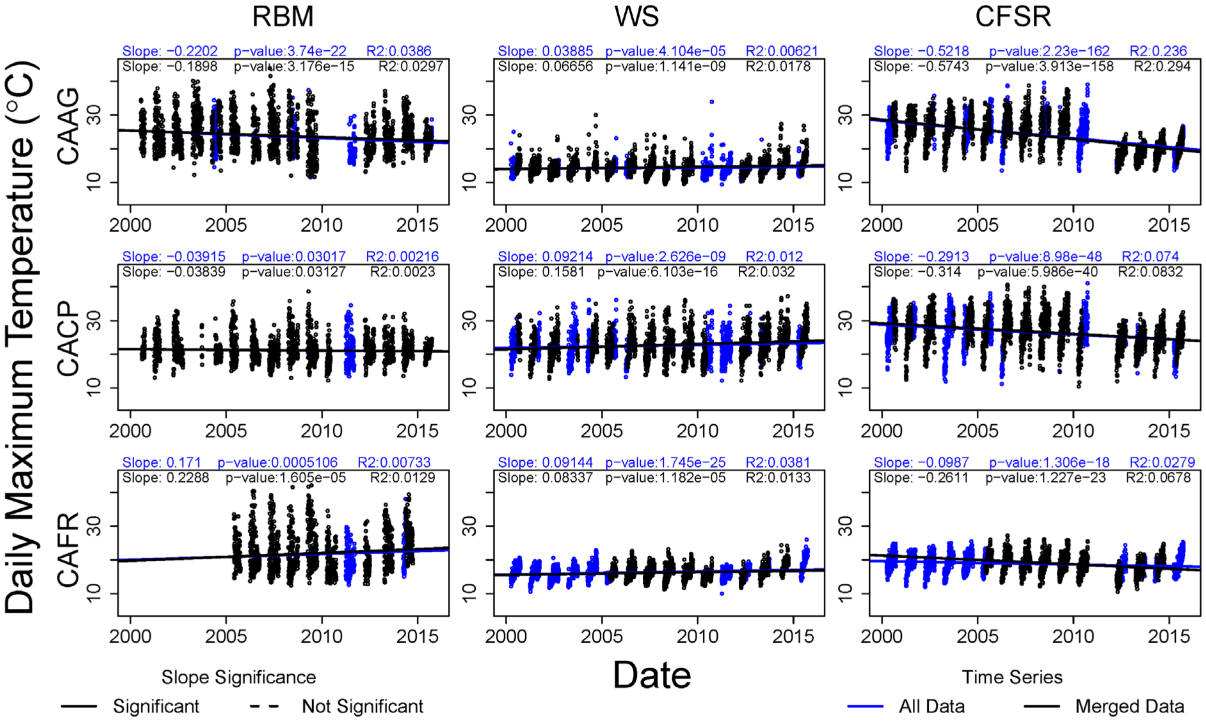

Figure S1.3: Daily maximum temperature time series (black; columns) for Robomussel (RBM; left), Weather Station (WS; center), and modeled (CFSR; right) datasets at three unique sites (rows). Trend lines are generated from simple linear regressions (OLS) run on data simultaneously available at all three data sources (black). For comparative purposes, additional data points and respective trend lines available for each data source (blue) are also shown. Associated R^2 values, p-values, and slopes (°C/year; dashed line if not significant, solid line if significant) are noted at the top of each panel. Sites are ordered latitudinally from north (top) to south (bottom).

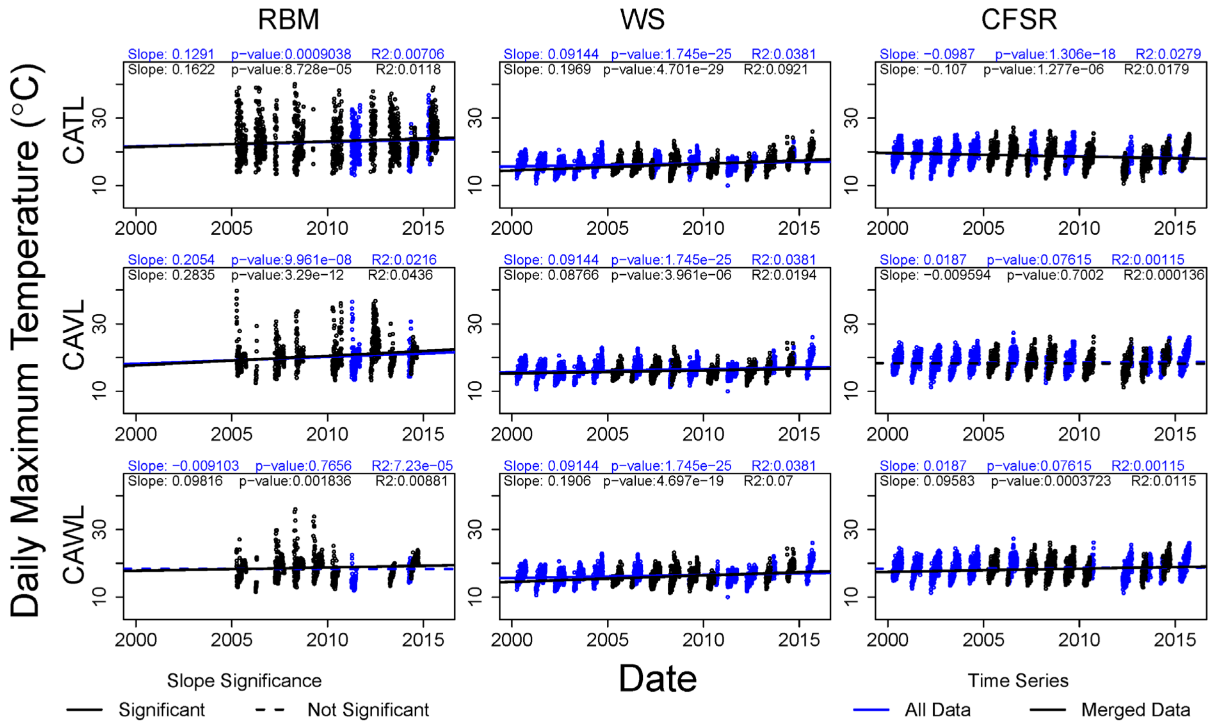

Figure S1.4: Daily maximum temperature time series (black; columns) for Robomussel (RBM; left), Weather Station (WS; center), and modeled (CFSR; right) datasets at three unique sites (rows). Trend lines are generated from simple linear regressions (OLS) run on data simultaneously available at all three data sources (black). For comparative purposes, additional data points and respective trend lines available for each data source (blue) are also shown. Associated R^2 values, p-values, and slopes (°C/year; dashed line if not significant, solid line if significant) are noted at the top of each panel. Sites are ordered latitudinally from north (top) to south (bottom).

Table S2: The rate of change (slope; °C /year) and associated standard errors generated by ordinary least squares (OLS) and quantile regressions (Q50: 50th percentile, $\tau = 0.50$ and Q90: 90th percentile, $\tau = 0.90$) run on Robomussel (RBM) temperature time series at all 12 sites (rows) from 2000-2015. Significance ($p < 0.05$) is indicated with an asterisk. Blue (red) cells represent positive (negative) trends over time. The rows represent the twelve sites in latitudinal order from Oregon (i.e., north; top) to Southern California (i.e., south; bottom).

Site	OLS	Q50	Q90
**ORBB**	0.207* ± 0.032	0.249* ± 0.049	0.152* ± 0.057
**ORSH**	0.147* ± 0.024	0.132* ± 0.030	0.200* ± 0.074
**CABD**	0.106* ± 0.029	0.123* ± 0.040	0.160* ± 0.078
**CAHS**	0.038 ± 0.021	0.049 ± 0.029	-0.012 ± 0.049
**CALL**	-0.165* ± 0.023	-0.102* ± 0.033	-0.423* ± 0.048
**CALS**	0.481* ± 0.054	0.675* ± 0.059	0.231 ± 0.149
**CAAG**	-0.190* ± 0.023	-0.168* ± 0.033	-0.337* ± 0.042
**CACP**	-0.038* ± 0.018	0.016 ± 0.018	-0.194* ± 0.057
**CAFR**	0.229* ± 0.053	0.268* ± 0.068	0.079 ± 0.148
**CATL**	0.162* ± 0.041	0.211* ± 0.049	-0.024 ± -0.103
**CAVL**	0.284* ± 0.040	0.185* ± 0.030	0.867* ± 0.155
**CAWL**	0.098* ± 0.031	0.110* ± 0.036	0.127* ± 0.051
